# Daydream Believer: Rumination, Self-Reflection and the Temporal Focus of Mind Wandering Content

**DOI:** 10.5964/ejop.v13i4.1425

**Published:** 2017-11-30

**Authors:** Daisy Shrimpton, Deborah McGann, Leigh M. Riby

**Affiliations:** a Department of Psychology, Northumbria University, Newcastle upon Tyne, United Kingdom; Webster University Geneva, Geneva, Switzerland

**Keywords:** rumination, self-reflection, mind-wandering, day-dreaming, sustained attention, self-generated thought

## Abstract

Current research into mind-wandering is beginning to acknowledge that this process is one of heterogeneity. Following on from previous findings highlighting the role of self-focus during mind wandering, the present study aimed to examine individual differences in rumination and self-reflection and the impact such styles of self-focus may have on mind-wandering experiences. Thirty-three participants were required to complete the Sustained Attention Response Task (SART), aimed at inducing mind-wandering episodes, whilst also probing the content of thought in terms of temporal focus. Self-report questionnaires were also administered after the SART to measure dispositional differences in style and beliefs regarding mind-wandering and assessments of individual differences in rumination and self-reflection. Those individuals with reflective self-focus showed a strong positive association with positive and constructive thoughts. Critically, ruminative self-focus was positively associated with a tendency for the mind to wander towards anguished fantasies, failures and aggression, but it was also positively associated with positive and constructive thoughts. Furthermore, while dispositional differences in self-focus showed no relationship with the temporal perspective of thoughts when probed during a cognitive task, performance on the task itself was related to whether participants were thinking about the past, present or future during that activity. Such findings are discussed in line with previous research, and provide a further step towards accounting for the heterogeneous nature of mind-wandering.

Human thinking is not always generated from the current external world, and often becomes focused internally on thoughts and feelings unrelated to the present moment. This state of mind-wandering, often defined as stimulus-independent thought, is experienced by all individuals, occupying as much as fifty per cent of our waking life (Killingsworth & Gilbert, 2010; Singer & McCraven, 1961).

Previous research into mind-wandering has focused on why mind-wandering occurs in the first instance, and what potential outcomes it may have for the individual. With regards to the former, three key theoretical viewpoints have been proposed to explain the onset and frequency of mind-wandering. The distractibility account proposes that task unrelated thought reflects a failure to deal with distraction regardless of whether this distraction stems from the external environment or is self-generated (Baird et al., 2012; Barron, Riby, Greer, & Smallwood, 2011; Shaw & Giambra, 1993). Conversely, the executive-function account has suggested that mind-wandering reflects a specific failure in executive control whereby individuals have impairment in processing task-relevant material and experience issues with inhibiting responses to irrelevant stimuli (McVay & Kane, 2010). In line with this view, when mind wandering episodes dominate, additional executive processing mechanisms are thought to be required for successful task completion (Riby, Smallwood, & Gunn, 2008). Finally, the decoupling account proposes that mind-wandering involves a dissociation in processing whereby the mind-wanderer’s attention becomes concentrated towards internal inner thoughts and feelings and disengaged from current external events in the environment (Smallwood, 2010; Smallwood et al., 2003).

Furthermore, past research has also began to establish what adaptive purpose mind-wandering may serve given how much time humans spend engaged in this process. On the one hand, research has revealed a number of positive outcomes, including the ability to mentally time travel (and thus maintain a coherent sense of self-identity), the ability to plan and prepare for the future, to manage long term personal goals and importantly such thoughts have been related to creative ability (Baird, Smallwood, & Schooler, 2011; Baird et al., 2011; Smallwood, Nind, & O’Connor, 2009; Smallwood, Ruby, & Singer, 2013; Suddendorf & Corballis, 2007; Tulving, 1987). On the other hand, alternative lines of research have consistently revealed decrements in task performance as a result of mind wandering including impacts on reading comprehension, signal detection, memory and performance on tasks of sustained attention and response inhibition (McVay & Kane, 2012; Smallwood, Baracaia, Lowe, & Obonsawin, 2003; Smallwood, Beach, Schooler, & Handy, 2008; Smallwood et al., 2004; Smallwood, McSpadden, Luus, & Schooler, 2008; Smallwood, McSpadden, & Schooler, 2008). Findings also suggest that this process comes at a detrimental cost to mood (Killingsworth & Gilbert, 2010; Smallwood, Fitzgerald, Miles, & Phillips, 2009; Smallwood, O’Connor, Sudbery, & Obonsawin, 2007).

Based on such mixed findings, recent suggestions have postulated that mind-wandering is a highly heterogeneous cognitive process with variable outcomes (Smallwood & Andrews-Hanna, 2013). It has further been suggested that understanding thought content is critical when considering the variable costs and benefits associated with mind-wandering (e.g., Andrews-Hanna et al., 2013). Thus, whilst previous research has been valuable for providing insight into how and why mind-wandering occurs, it fails to offer any understanding of how content may mediate variable mind-wandering outcomes. In view of this, it is now important for research to focus on the content of the mind-wandering experience and consider factors which may impact upon the nature of these thoughts.

One factor to consider when explaining the heterogeneity observed in previous research is the temporal focus of mind-wandering. Given how frequent prospective orientation is during episodes, it has previously been suggested that a focus on the future must be adaptive in some way, perhaps by facilitating the planning and preparation of future events (Baird, Smallwood, & Schooler, 2011; Riby et al., 2014; Schacter, Addis, & Buckner, 2007). Conversely, a retrospective focus does not appear to facilitate the processing of personal goals and a general over-focus on the past has been associated with symptoms of depression (Baird, Smallwood, & Schooler, 2011; Nolen-Hoeksema, Wisco, & Lyubomirsky, 2008). Furthermore, links have been made between retrospective mind-wandering and low mood (Poerio, Totterdell, & Miles, 2013; Smallwood & O’Connor, 2011).

This variability in the temporal focus of mind-wandering has been partially linked to individual differences in mind-wandering content in an influential paper by Singer (1975). He distinguished between three differing daydreaming styles which may provide some preliminary insight into the mechanisms involved during different mind-wandering episodes. The positive-constructive day-dreaming style, characterised by an acceptance of daydreaming, involving pleasant and future focused thoughts and the generation of original ideas may reflect findings related to more adaptive mind-wandering outcomes, such as future planning and creativity. By contrast, the guilt and fear of failure daydreaming style, is characterised by anguished fantasies about the past and future, is often depressing and frightening and with an element of panic. Finally, the poor attentional control daydreaming style, may reflect maladaptive mind-wandering outcomes, such as low mood and cognitive failure.

The value of considering content of mind-wandering episodes has also been directly highlighted by Andrews-Hanna et al. (2013). In such research, the content of mind-wandering was examined for variables such as valence, specificity and self-relevance. It was shown that individuals who rated their thoughts as more negative and more personally significant scored higher on measures of depression and trait negative affect. Conversely, those who rated their thoughts as less specific scored higher on constructs linked to rumination, whilst individuals who viewed their thoughts as more positive, less personally significant and more specific yielded high scores on measures associated with increased wellbeing. Researchers concluded that considering the content of mind-wandering episodes can account for some of the variability documented in previous research.

When considering factors that might influence differing mind-wandering content, recent research has begun to examine in greater detail the tendency of mind-wandering to be internally orientated in terms of its focus (Baird, Smallwood, & Schooler, 2011). Marchetti, Koster, Sonuga-Barke, and De Raedt (2012) suggest that this internally-orientated focus is associated with greater self-focus during these episodes, the nature of which may vary. Specifically, two distinct types of self-focus are frequently identified, rumination and reflective self-focus (Trapnell & Campbell, 1999). Whilst rumination is defined as negatively evaluative and judgmental in nature with a passive dwelling on personal concern, self-reflection is characterised as an epistemic curiosity towards the self, allowing individuals to openly explore their inner experiences (Trapnell & Campbell, 1999). Despite the lack of direct research examining these two distinct types of self-focus on mind-wandering, past research has suggested that they could each have a significant impact on its content.

Previous research has made associations with rumination and an over-focus on the past, pessimistic expectancies for the future, and more negatively toned thought content (Lavender & Watkins, 2004; Lyubomirsky & Nolen-Hoeksema, 1995; Nolen-Hoeksema, Wisco, & Lyubomirsky, 2008). Research directed more so to mind-wandering has also drawn links between rumination and dysphoria experienced as a result of a wandering mind (Smallwood & O’Connor, 2011). It has also been found that internal focus during mind-wandering is associated with a higher level of rumination, which was subsequently associated with a temporary reduction in mood (Marchetti, Koster, & De Raedt, 2013). Furthermore, research has theoretically implicated the role of rumination as a mediator between low mood and a retrospective bias during mind-wandering, based on the notion that this style of self-focus often involves passively dwelling on experiences (Smallwood & O’Connor, 2011). In view of research documenting certain cognitive decrements associated with mind-wandering, research has also documented a relationship between rumination and impaired concentration on academic tasks (Lyubomirsky, Kasri, & Zehm, 2003).

Conversely, research into self-reflection has been associated with reduced levels of depression when contrasted with its counterpart rumination, increased creativity and the formation of goals (Takano & Tanno, 2009; Koestner, Lekes, Powers, & Chicoine, 2002; Verhaeghen, Joormann, & Aikman, 2014). Research directly examining self-reflection alongside the mind-wandering experience has also demonstrated that a period of self-reflection can lead to a prospective bias during mind-wandering. It was suggested in such research that this style of self-focus is of importance for stimulus independent thinking which is directed toward the future (Smallwood et al., 2011).

Based on these findings, it is clear that individual differences in style of self-focus are likely to impact upon the content of thought and the temporal focus during mind-wandering. As such, the current study aims to investigate whether individual differences relating to rumination and self-reflection are associated with specific content or temporal orientation when probed during mind-wandering. It is hoped that this research will begin to suggest potential mechanisms involved in variable mind-wandering episodes. It is hypothesised that rumination will be positively associated with past temporal focus when mind-wandering thoughts are probed during a cognitive task. Furthermore, rumination will be positively related to dispositional tendencies toward mind-wandering associated with guilt and fear of failure as well as poor attentional control. It is further hypothesised that self-reflection will be positively associated with a future temporal focus when thoughts are probed and a general tendency toward mind-wandering that is positive and constructive in content.

Based on findings from previous research revealing the impact of mind wandering on cognitive performance (including sustained attention, response inhibition and signal detection), a secondary concern of the current study is to examine the relationship between the content of mind-wandering and cognitive performance on a sustained attention task (Smallwood, Beach, Schooler, & Handy, 2008; Smallwood et al., 2004; Smallwood, McSpadden, & Schooler, 2008). Furthermore, the relationship between cognitive task performance and style of self-focus will also be explored. It is expected that task performance will be negatively associated with guilt-fear of failure mind-wandering, poor attentional control, past temporal focus, and rumination (Lyubomirsky, Kasri, & Zehm, 2003). By contrast, task performance will be positively associated with positive constructive mind-wandering, present temporal focus, and self-reflection.

## Method

### Design

The study employed a correlational design with inclusion of the following variables; scores for the two RRQ subscales (including the rumination subscale and the self-reflection sub-scale), scores on the three distinct day-dreaming styles (positive constructive day-dreaming, guilt-fear-of failure day-dreaming and poor attentional control), temporal focus (indexed by the percentage of responses indicating participants were past, present or future focused when probed during the SART) and aspects of performance measured during the sustained attention response task (SART). That is, reaction time (RT), accuracy and the number of false alarms.

### Participants

An opportunity sample of 17 males (mean age = 36.71, *SD* = 22.22) and 16 females (mean age = 47.88, *SD* = 17.52) between the ages of 18-82 participated in the study. Participants were recruited from areas within the North East of England through advertisements on social media websites. The study was advertised as research into differing types of self-focus employed during a computerised task. No compensation was offered for participation. Individuals who were interested in participating were required to contact the researcher for further information. The exclusion criteria was kept as inclusive as possible and thus only excluded any individual under the age of 18. Thirty-three participants were recruited in total, however there were instances whereby participants did not provide complete data for every measure included. The participants who had provided incomplete data on some of the measures were still included in the study and as such sample sizes for each individual correlation range from 31 to 33.

### Sample Size Considerations

Given the relative novelty of current research it was difficult to ascertain an effect size and carry out a power analysis. However, research from our lab (Martinon et al., 2017) considering similar issues between daydreaming frequency and rumination demonstrated a Cohen’s *d* = 0.98. Given this effect size, a sample of 30 is appropriate with a power of 0.8.

### Materials

#### Sustained Attention Response Task

The study employed the Sustained Attention Response Task (SART; Robertson, Manly, Andrade, Baddeley, & Yiend, 1997). Participants were required to respond as quickly as possible to frequent and relevant stimuli, whilst inhibiting their responses to infrequent stimuli. The program included X and Y letter stimuli, both of which followed a cross hair to ensure that visual alignment was focused upon the stimuli. The letters X and Y were identical in size and colour, and were presented on the same white background. The program was modified to ensure that the task would increase the likelihood of mind-wandering by using a 3000ms delay in between stimuli based on past research utilising a similar delay (Birnie, Smallwood, Reay, & Riby, 2015). Prior to the task, participants were presented with the following instructions: “In this task you will see the letters X and Y appear on the screen. Your task will be to push the space bar whenever you see the letter X. Do nothing when the letter Y appears on the screen”. Participants were initially allowed a practice phase to ensure that they understood the task instructions. During this practice phase, 10 sample stimuli were presented in a 9/1 ratio (X/Y), with stimuli occurring 3000 milliseconds apart. Once the practice phase was complete, the testing condition begun. This condition consisted of 13 blocks, with 20 stimuli presented in each block at a 16/4 ratio (X/Y), with stimuli occurring 3000 milliseconds apart from each other. Between each block, participants were presented with thought probes to assess the temporal focus of mind-wandering. Each participant was required to respond to the following question: “Did your mind wander? During the previous responses were you 1) Thinking about the past 2) Thinking about the ‘here and now’ 3) Thinking about the future”. Participants were asked to respond using the corresponding letters on the keyboard.

#### Rumination-Self-Reflection Questionnaire (RRQ)

The RRQ (Trapnell & Campbell, 1999) was used to measure dispositions of both rumination and self-reflection. The measure was suitable for the current study as it included two sub-scales (with 12 items for each) measuring the two relevant theoretical constructs. The first 12 items assessed self-reflection, including items such as “I love exploring my “inner” self”. In contrast, the next 12 items assessed rumination, including items such as “I tend to ruminate or dwell over things that have happened to me for a long time afterwards”. Below each statement participants were provided with a Likert scale from 1-5 with 1 being strongly disagree and 5 being strongly agree. The meanings of each number were explained before the questionnaire statements. Participants were required to indicate their level of agreement or disagreement to each statement by circling one of the scale categories. High scores on each subscale were indicative of high dispositions of self-reflection and rumination respectively. Trapnell and Campbell (1999) originally documented alpha estimates of reliability exceeding .90 for both sub-scales, as such the measure is considered to have excellent internal consistency. Consistent with this, Cronbach’s alpha was high for both the rumination and self-reflection subscales in the current study, αs = .90 and .89 respectively.

#### The Short Imaginal Processes Inventory (SIPI)

The Short Imaginal Processes Inventory (SIPI; Singer, Aneshensel, & Antrobus, 1982) was used to assess styles of day-dreaming employed by the individuals as part of their daily functioning. The SIPI was appropriate for the current study as it consisted of three relevant theoretical constructs corresponding to participant’s inner experiences. The SIPI scored participants on three subscales, with 15 items for each respective category. Subscales included- 1) positive-constructive daydreaming – characterised by the belief that daydreams are worthwhile, solve problems, help generate original ideas, are stimulating, generate pleasant thoughts and present a high degree of future orientation - 2) guilt-fear of failure dreaming, characterised by depressing and frightening daydreams with panicky overtones and - 3) poor attentional control, characterised by tendencies toward mind wandering and drifting thoughts due to boredom or a poor attention span (Singer, 1975). The measure consists of statements such as “I am the kind of person whose thoughts often wander”. Each item was followed by a Likert scale in the form of 5 boxes, numbered from 1 to 5 with 5 indicating “very true or strongly characteristic of me”, and 1 indicating “definitely untrue or strongly uncharacteristic of me”. Participants were provided with the following instructions prior to completing the questionnaire: “Each statement says something about daydreams or daydreaming, indicate to what extent each statement applies to you or is true for you, by checking the box above the appropriate number”. The scale ranging from 1-5 was presented below these instructions and appeared on every subsequent page of the questionnaire to remind participants which numbers corresponded to each level of agreement. High scores on each daydreaming category were indicative of high levels of the respective daydreaming style they aimed to measure. Singer et al. (1982) reported good internal consistency for the positive-constructive daydreaming, the guilt-fear of failure, and the poor attentional control subscales (αs = .80, .82, and .83, respectively) and adequate reliability was shown for these subscales in the current sample (αs = .75, .65, and .74, respectively).

### Procedure

Ethical approval was received from the Ethics Committee at the Department of Psychology, Northumbria University. Each participant attended a quiet and controlled office for testing to take place whereby only the researcher and the participant were present. Participants were initially provided with the participant information sheet and subsequently provided their informed consent. Given that there is no standard order for the self-report measures and the SART, judgement regarding order was made based on the notion that providing participants with the self-report measures prior to the SART may have inadvertently induced particular mood states which could have subsequently impacted responses to thought probes. Furthermore, for similar reasons self-report measures and the SART were not counterbalanced. Consenting participants were then given initial instructions with regards to the SART task which involved providing examples of the thought probes to ensure that participants fully understood the meaning of these questions. Participants were then asked to follow the instructions on the screen in order to complete the SART, which took approximately 15 minutes. Upon completion, participants were administered a brief demographic questionnaire, which asked them to state their age and gender, as well as the RRQ and the SIPI. Completed questionnaires were then handed back to the researcher and participants were provided with a debrief form and given the opportunity to ask the researcher any questions with regards to the study. The whole procedure took approximately 30 minutes. Participants who expressed an interest to be informed of the results upon completion were subsequently emailed with a feedback sheet once data analysis had taken place.

## Results

### Analytical Strategy

#### Self-Reflection and Rumination

The impact of day dreaming in everyday life in relation to relatively more positive (self-reflection) and negative (rumination) thoughts were considered first. A series of one tailed correlations were carried out on 1) rumination scores in relation to temporal focus (past, present and future) and the styles of daydreaming elicited by the SIPI 2) self-reflection scores in relation to temporal focus (past, present, future) and the styles of daydreaming elicited by the SIPI. Together these first analyses will contribute to our understanding the *content* of relatively more positive self-reflection and relatively more negative ruminations.

#### Behavioural Performance

Subsidiary data analyses were carried out to further characterise the nature of the mind wandering episodes and importantly how different ‘types’ have a cost or benefit to cognitive performance. A series of one tailed correlations were carried out on SART task performance (indexed via accuracy, RT and false alarms) and 1) SIPI factors, 2) the temporal focus (past, present and future) of thoughts and 3) the self-reflection and rumination factors.

#### Rumination Factors on Temporal Focus

Inconsistent with the first hypothesis, no significant relationship was identified between rumination scores and a past temporal focus, *p* > .05. All other relationships between rumination scores and temporal focus were also non-significant, *ps* > .05.

#### Rumination Factors on Content

**Figure 1:**
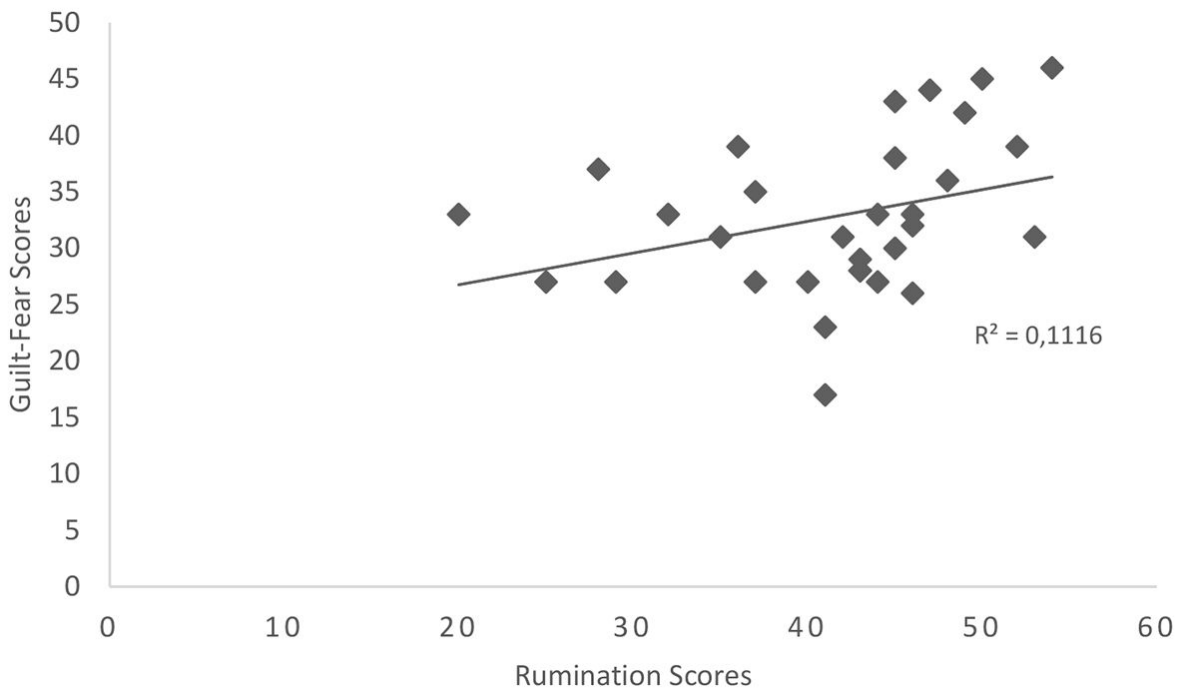
Scatterplot showing the relationship between rumination scores and scores on the guilt-fear of failure day-dreaming category, *N* = 31.

**Figure 2:**
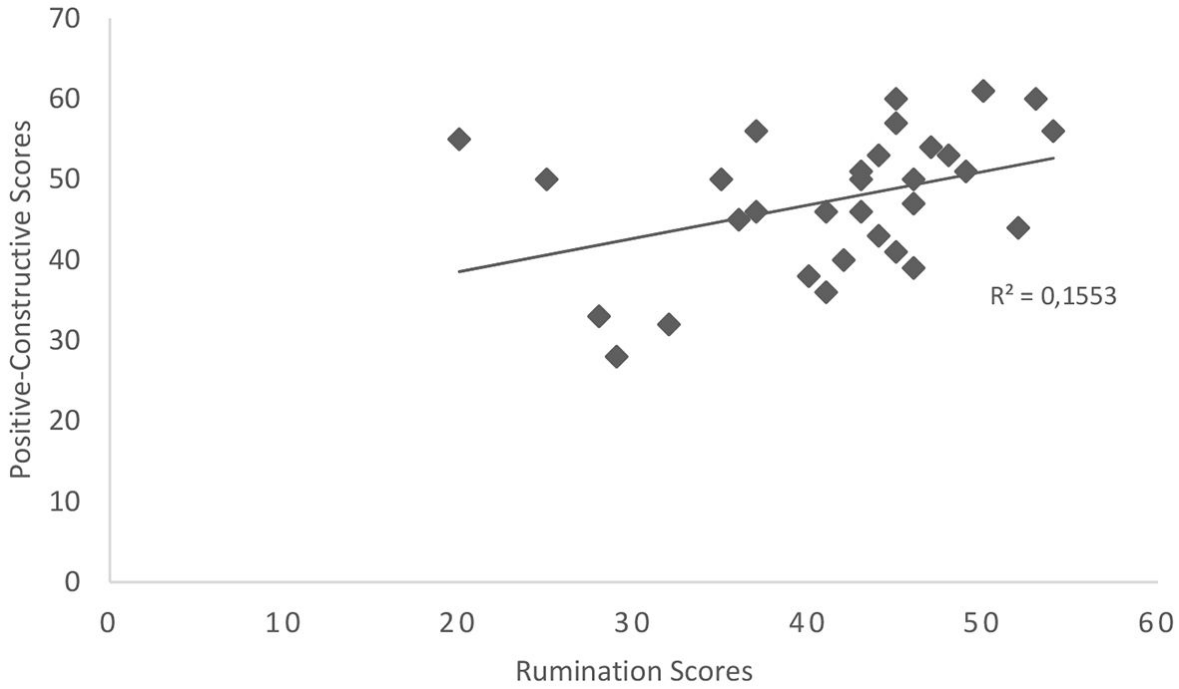
Scatterplot showing the relationship between rumination scores and scores on the positive-constructive day-dreaming category, *N* = 31.

In line with the second hypothesis, a positive relationship was identified between rumination scores and scores for the guilt-fear of failure category which was significant, *r* = .334, *p* = .033 (see Figure 1). Surprisingly, a positive relationship was also identified between rumination scores and scores for the positive-constructive daydreaming style which was also significant, *r* =.394, *p* = .028, (see Figure 2). Inconsistent with the third hypothesis, no significant relationship was identified between rumination scores and scores for the poor attentional control category, *p* > .05.

#### Self-Reflection on Temporal Focus

Inconsistent with the fourth hypothesis, no significant relationship was identified between self-reflection scores and a future temporal focus, *p* > .05. No other significant relationships were identified between self-reflection scores and temporal focus, *ps* > .05.

#### Self-Reflection on Content

**Figure 3:**
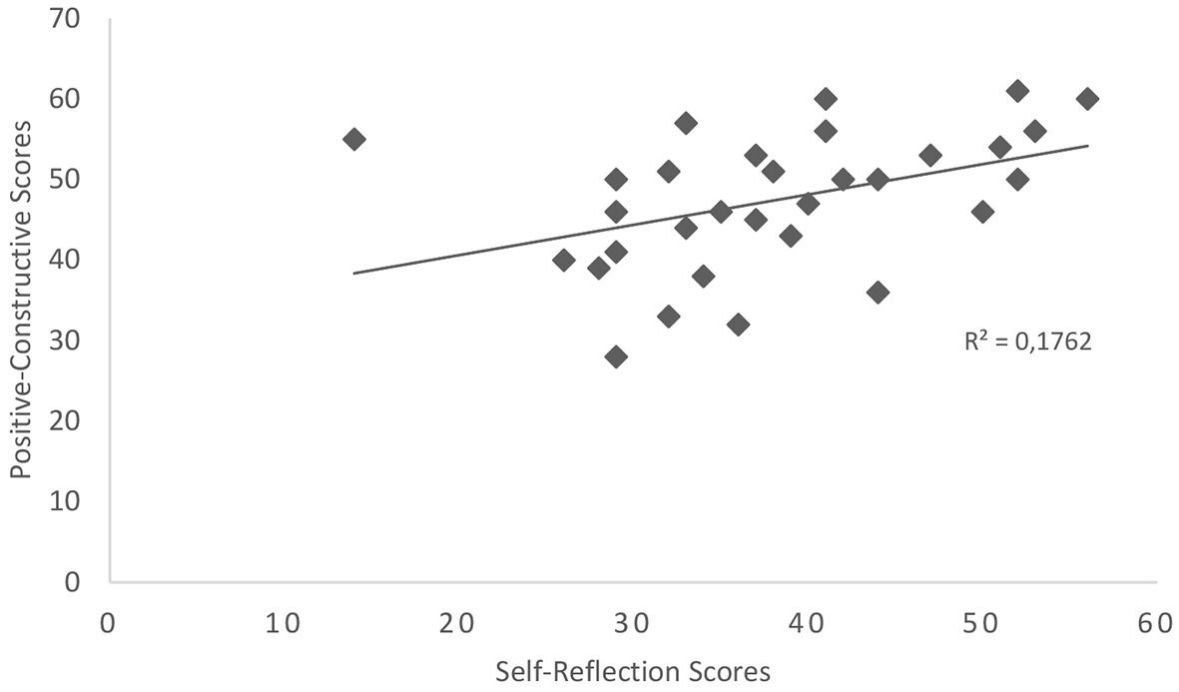
Scatterplot showing the relationship between self-reflection scores and scores on the positive-constructive day-dreaming category, *N* = 31.

In line with the fifth hypothesis, a positive relationship was found between self-reflection scores and scores on the positive-constructive category which was significant, *r* = .420, *p* = .009 (see Figure 3). No significant relationships were identified between self-reflection scores and scores relating to the guilt-fear of failure and poor attentional control daydreaming categories, *ps* > .05.

### Subsidiary Analyses

#### SIPI Factors and SART Task Performance

Inconsistent with the second set of hypotheses, no significant relationships were identified between scores on the positive constructive day-dreaming category, poor attentional control category or the guilt-fear of failure category and performance during the SART, *ps* > .05.

#### Temporal Focus and SART Task Performance

Results concerning the temporal focus and task performance offer partial support for the second set of hypotheses. A significant negative relationship was identified between the percentage of responses indicating participants were past focused and RT, *r* = -.510, *p* = .002. A positive relationship was also identified between the percentage of responses indicating participants were past focused and the number of false alarms made during the SART, *r* = .544, *p* = .001, However, no significant relationship was identified between past focus and accuracy, *p* > .05.

A significant, negative relationship was identified between the percentage of responses indicating participants were present focus and number of false alarms made during the SART, *r* = -.360, *p* = .023. However, no significant relationships were identified between a present temporal focus and RT or accuracy, *p* > .05.

No significant relationships were identified between future focus and performance during the SART, *p* > .05.

#### Self-Reflection/Rumination Factors and SART Task Performance

Inconsistent with the second set of hypothesis, no significant relationships were found between self-reflection and rumination scores and performance during the SART, *ps* > .05.

## Discussion

Current research examining mind-wandering has begun to acknowledge that this cognitive process is highly heterogeneous. It is assumed that through focusing on where in time individuals wander to during episodes and what individuals think about when off task will enable a greater understanding of the mechanisms driving variable outcomes associated with mind-wandering. The current study aimed to examine the relationships between individual differences in self-focus (rumination vs. self-reflection) on the content and temporal focus of mind-wandering episodes.

In line with predictions, when mind-wandering content was measured in terms of dispositional, everyday daydreaming style, self-reflection was found to be positively associated with a positive-constructive daydreaming style typically characterised by pleasant thoughts. A self-reflective style of self-focus allows individuals to openly explore their inner feelings in a positive light, with a sense of acceptance (Trapnell & Campbell, 1999). Thus, it is plausible that this process of inner exploration could subsequently give rise to this more positive and constructive daydreaming style. Support for this postulation can be drawn from past research highlighting a number of positive outcomes associated with periods of self-reflection, that are also in accordance with the beneficial outcomes of mind-wandering including creativity, goal planning, future planning and happiness (Bryant, Smart, & King, 2005; Koestner, Lekes, Powers, & Chicoine, 2002; Smallwood et al., 2011; Verhaeghen, Joormann, & Aikman, 2014). These results may be valuable in future research establishing how individuals may maximize more positive mind-wandering episodes, in line with the content regulation hypothesis (Smallwood & Andrews-Hanna, 2013). As such, with further research this finding may offer some important insight into mechanisms driving more positive mind-wandering experiences, such as those yielding the adaptive outcomes observed in previous research.

Consistent with hypotheses, research also revealed a positive relationship between high levels of rumination and Singer’s (1975) guilt-fear of failure daydreaming style (characterised by anguished fears, fantasies and aggression). This finding can be reconciled with previous research into the role of rumination, with associations between rumination and more negatively toned thought content (Nolen-Hoeksema, Wisco, & Lyubomirsky, 2008). Furthermore, findings are also in line with recent research revealing an association between ruminators and the propensity for negative mind-wandering (Hoffmann, Banzhaf, Kanske, Bermpohl, & Singer, 2016). Thus, the present findings may offer further explanation for the association between mind-wandering and negative mood identified in previous research, with rumination acting as a mediator in this process (Smallwood & O’Connor, 2011). Somewhat surprisingly, high levels of rumination were also positively associated with Singer’s (1975) positive constructive daydreaming style. This appears contradictory when considering that positive-constructive daydreaming is often characterised by the generation of pleasant thoughts (Singer, 1975). However, along with the generation of pleasant thought content, the positive-constructive daydreaming style is also characterised by the belief that daydreams are worthwhile, solve problems and help generate original ideas. Researchers have suggested that this finding may be due to how the SIPI assesses positive-constructive daydreaming. For example, many of the questions relating to this measure focus on the attitudes individuals have with regards to their own inner experiences. Thus, it may be that ruminators view their inner experiences as more constructive in nature, despite thoughts generally being negatively toned in terms of content. This is consistent with previous research suggesting that individuals view their ruminative thoughts as being useful, believing that this style of thinking helps them solve problems or make sense of past events, regardless of how much of a detriment it is to overall wellbeing (Watkins & Baracaia, 2001).

In terms of the temporal focus of mind-wandering, current findings did not support the hypothesis that there would be a positive relationship between self-reflection and a prospective temporal focus. Previous research has suggested that this style of self-focus may be vital for future thinking during mind-wandering, which in turn facilitates beneficial outcomes (Smallwood et al., 2011). In such research, self-reflection was induced rather than measured at a dispositional level. Inconsistencies can perhaps therefore be explained through differing methodology. The mismatch between methods probing the wandering mind is clearly critical. The fact that we used a mixture of self-report and online measures could offer an explanation as to why we weren't able to replicate this finding.

Contrary to predictions, rumination was not positively associated with a retrospective temporal focus. These non-significant findings appear inconsistent in light of previous research suggesting that rumination is often associated with an over-focus on the past (Nolen-Hoeksema, Wisco, & Lyubomirsky, 2008). Furthermore, findings are also inconsistent with research directly examining mind-wandering experiences in ruminators, revealing an association between ruminators and the propensity for retrospective mind-wandering (Hoffmann, Banzhaf, Kanske, Bermpohl, & Singer, 2016). Previous research has also speculated that rumination may account for the relationship between low mood and a retrospective bias during mind-wandering (Smallwood & O’Connor, 2011). One suggestion for this inconsistency may be due to low levels of meta-consciousness in ruminators. Specifically, individuals identified as ruminators may have some level of awareness that they were not task focused when their thoughts are probed but experience difficulty when required to pinpoint where in time their thoughts were orientated towards. This suggestion is supported by previous research revealing that those who are less mindful (such as those who frequently ruminate) are less aware of their stimulus independent or task unrelated thoughts (Stawarczyk, Majerus, Van der Linden, & D’Argembeau, 2012). Alternatively, given that Hoffmann and colleagues (2016) were able to identify a signification relationship between rumination and a retrospective focus, it may be the case that ruminators within the current study do not typically ruminate to the same extent as participants taking part in previous research, such as those with a diagnosis of major depressive disorder (i.e. Hoffmann et al., 2016).

Current research also aimed to examine relationships between mind-wandering characteristics and cognitive task performance. It was expected that, whilst task performance would be negatively associated with guilt-fear of failure mind-wandering and poor attentional, it would show a positive relationship with positive and constructive thoughts. Contrary to such predictions, dispositional differences in mind-wandering style showed no relationship with cognitive task performance. Similarly, no relationship between style of self-focus and task performance was observed. Specifically, the predicted negative association between rumination and task performance was not observed, nor was the positive association between self-reflection and performance on the task. One possible explanation for the lack of significance is that individual differences measures asked participants to report their *typical* thinking and daydreaming styles, but these may not be accurate reflections of what participants were actually thinking about during the task itself. It has been suggested that the sampling method of probing the content of thoughts while participants are performing a task provides a more online measure of the content of mind-wandering. In view of this, future research could benefit from examining whether online probes into the emotional valence of mind-wandering thought using the experience-sampling method provide better predictors of task performance. Furthermore, it is worth also directing future research towards whether measures of dispositional mind-wandering style are better at predicting outcomes when participants are asked to self-report on their typical levels of performance and mood.

This suggestion is further supported in that in the current study, online probes into the content of mind-wandering thoughts were measured in relation to their temporal perspective. When mind-wandering content was measured in this way, relationships between mind-wandering content and task performance were observed. Specifically, it was found that reports of being focused on the past were positively associated with false alarm responses to the stimuli and negatively associated with reaction time. However, when participants reported that they were focused on the present they were less likely to make false alarm responses. One explanation for this is that when participants were mind-wandering about thoughts related to the past they were mindlessly pressing the response keys at a more rapid rate and were less focused on the status of the stimuli that were presented to them. Consequently, where individuals were dedicating attentional resources to task unrelated past thoughts, effectively inhibiting a response to the infrequent stimuli became problematic. Although accuracy did not improve when participants reported they were focused on the here and now, they were better at inhibiting a response when it was appropriate to do so (see Greer at al., 2013).

The findings of the current study have a number of important implications, particularly in relation to how researchers should investigate mind-wandering in the future. It is clear that mind-wandering is a complex phenomenon, influenced by a number of interconnected factors. As such, current research supports the suggestion that researchers should now focus their attention towards the content of mind-wandering episodes, which may in turn facilitate a greater understanding of the processes associated with mind-wandering episodes (Smallwood & Andrews-Hanna, 2013). Furthermore, current research also holds important implications with regards to acknowledging individual differences in mind-wandering and that as a result of this, research findings should not be generalised across every person or situation.

Despite findings being a valuable addition to the current body of research into mind-wandering, there are a number of limitations that must be addressed in further investigations. The report effects sizes (*r*’s ranging from 0.34 – 0.42 for self-reflection/rumination analysis; 0.36 to 0.54 for mental performance analysis) here suggest future studies building on our findings need to be sufficiently powered to disentangle the processes engaged during mind wandering episodes. One limitation of the current study is that the content of mind-wandering in terms of positive versus negative valence was only measured as a dispositional trait, using a retrospective self-report questionnaire. Similarly, the content of mind-wandering in terms of temporal perspective was only investigated using online experience sampling thought probes. Future research should take a more systematic look at what these different methods of sampling thought content tell us about the factors that predict variable mind-wandering content, and the impact of this variable content on performance and well-being outcomes.
